# Comparison of three malnutrition assessment criteria for predicting postoperative complications and overall survival in colorectal cancer

**DOI:** 10.3389/fnut.2026.1996149

**Published:** 2026-10-28

**Authors:** Wei Wei, Yangming Li, Guiren Fang, Jinliang Jian, Zhenmeng Lin

**Affiliations:** NHC Key Laboratory of Cancer Metabolism, Fujian Cancer Hospital, Clinical Oncology School of Fujian Medical University, Fuzhou, China

**Keywords:** colorectal cancer, GLIM criteria, malnutrition, postoperative complications, survival

## Abstract

**Background:**

Malnutrition is highly prevalent in patients with colorectal cancer and is associated with adverse postoperative outcomes and shortened survival. However, the comparative predictive performance of commonly used nutritional assessment tools—PG-SGA, ESPEN, and GLIM criteria—remains unclear.

**Methods:**

A total of 1,258 patients who underwent radical resection for colorectal cancer were retrospectively enrolled. Malnutrition was diagnosed simultaneously using the PG-SGA, ESPEN, and GLIM criteria. Agreement among the three tools was evaluated using Cohen’s kappa coefficient. Multivariable logistic regression, an exploratory random forest analysis, and Cox proportional hazards models were applied. For the logistic regression models, ROC curve analysis and pairwise AUC comparisons using DeLong’s test were performed; for the Cox models, bootstrap-based C-index comparisons were conducted. Propensity score matching (PSM) was performed for the GLIM criterion as a *post hoc* sensitivity analysis to explore the association between GLIM-defined malnutrition and clinical outcomes.

**Results:**

The prevalence of malnutrition defined by PG-SGA, ESPEN, and GLIM was 24.7%, 15.7%, and 29.9%, respectively, with pairwise agreements ranging from moderate to substantial. All three criteria were independent risk factors for postoperative complications, with GLIM having the highest odds ratio (OR = 1.869, *p* < 0.001). AUC comparison further showed that the GLIM-incorporated model achieved the numerically highest AUC (0.781), with a significantly higher AUC than the ESPEN-based model (*p* = 0.023), whereas no significant difference was observed between GLIM and PG-SGA (*p* = 0.172). For OS, PG-SGA (HR = 1.364, *p* = 0.014) and GLIM (HR = 1.415, *p* = 0.004) were independent prognostic factors, whereas ESPEN was not (*p* = 0.129). Bootstrap C-index comparisons showed that the GLIM-based model provided modestly better prognostic discrimination than both the PG-SGA- and ESPEN-based models (both *p* < 0.05), although the absolute C-index improvement was small (approximately 0.015). After PSM balancing baseline characteristics, GLIM-defined malnutrition remained significantly associated with prolonged postoperative hospital stay, higher complication rates, and reduced 5-year overall survival.

**Conclusion:**

The three malnutrition diagnostic criteria are not interchangeable. For postoperative complications, GLIM demonstrated better discriminative ability than ESPEN but was comparable to PG-SGA. For long-term OS, GLIM provided modestly better prognostic discrimination than both PG-SGA and ESPEN, although the absolute improvement was small. These findings support the potential clinical utility of GLIM for perioperative nutritional risk assessment; however, the clinical relevance of these relatively small performance differences warrants further validation.

## 1 Introduction

Colorectal cancer ranks as the third most common malignancy and the second leading cause of cancer-related mortality worldwide, with its global burden continuing to rise—most notably among individuals under 50 years of age, in whom the incidence has been increasing at an alarming rate (1, 2). Surgical resection remains the cornerstone of curative-intent treatment. In recent years, advances in surgical techniques and perioperative management have substantially improved short-term outcomes for patients with colorectal cancer; nonetheless, postoperative complications and long-term survival remain central concerns in clinical practice (3–5).

Malnutrition is highly prevalent among patients with colorectal cancer, arising from a complex interplay of tumor-induced hypermetabolism, systemic inflammatory responses, reduced dietary intake, and gastrointestinal dysfunction (6, 7). This nutritional depletion impairs immune competence, compromises wound healing capacity, and diminishes physiological reserves, thereby predisposing patients to an elevated risk of postoperative complications and shortened survival (8–11). Currently, three nutritional assessment tools are commonly used in clinical practice: the Patient-Generated Subjective Global Assessment (PG-SGA), the criteria proposed by the European Society for Clinical Nutrition and Metabolism (ESPEN), and the Global Leadership Initiative on Malnutrition (GLIM) criteria. These instruments differ substantially in their diagnostic frameworks and threshold definitions. The PG-SGA is a multidimensional scale specifically designed for cancer patients, integrating patient-reported symptoms with clinician-rated physical examination (12, 13). In contrast, the ESPEN criteria rely primarily on objective anthropometric parameters, including body mass index and unintentional weight loss (14–16). Meanwhile, the GLIM criteria employ a two-step approach that combines phenotypic criteria (weight loss, low BMI, and reduced muscle mass) with etiologic criteria (reduced food intake, disease burden/inflammation) (17, 18).

Although each of these criteria has been validated individually, the diagnostic agreement among them and their comparative predictive performance for clinical outcomes in patients with colorectal cancer remain insufficiently evaluated (19–21). Therefore, this study aimed to compare the three criteria—PG-SGA, ESPEN, and GLIM—in terms of diagnostic consistency and predictive validity for postoperative complications and overall survival through a retrospective analysis of patients who underwent curative resection for colorectal cancer, with the goal of providing evidence-based information on the comparative performance of these tools in perioperative management.

## 2 Methods

### 2.1 Study population

We retrospectively reviewed the clinical data of patients who underwent radical resection for colorectal cancer at our institution between January 2018 and March 2020. Patients were included if they met the following criteria: (1) histopathologically confirmed primary colorectal adenocarcinoma; (2) underwent curative resection; (3) had complete preoperative clinical data, including demographic characteristics, medical history, laboratory findings, and nutritional assessment records; and (4) had complete postoperative follow-up data. Exclusion criteria were: (1) colorectal cancer with synchronous liver metastases; (2) emergency surgery; (3) recurrent colorectal cancer; and (4) open surgery. A total of 1,258 patients were ultimately enrolled in this study. The study flow diagram is presented in Figure 1.

**Figure 1:**
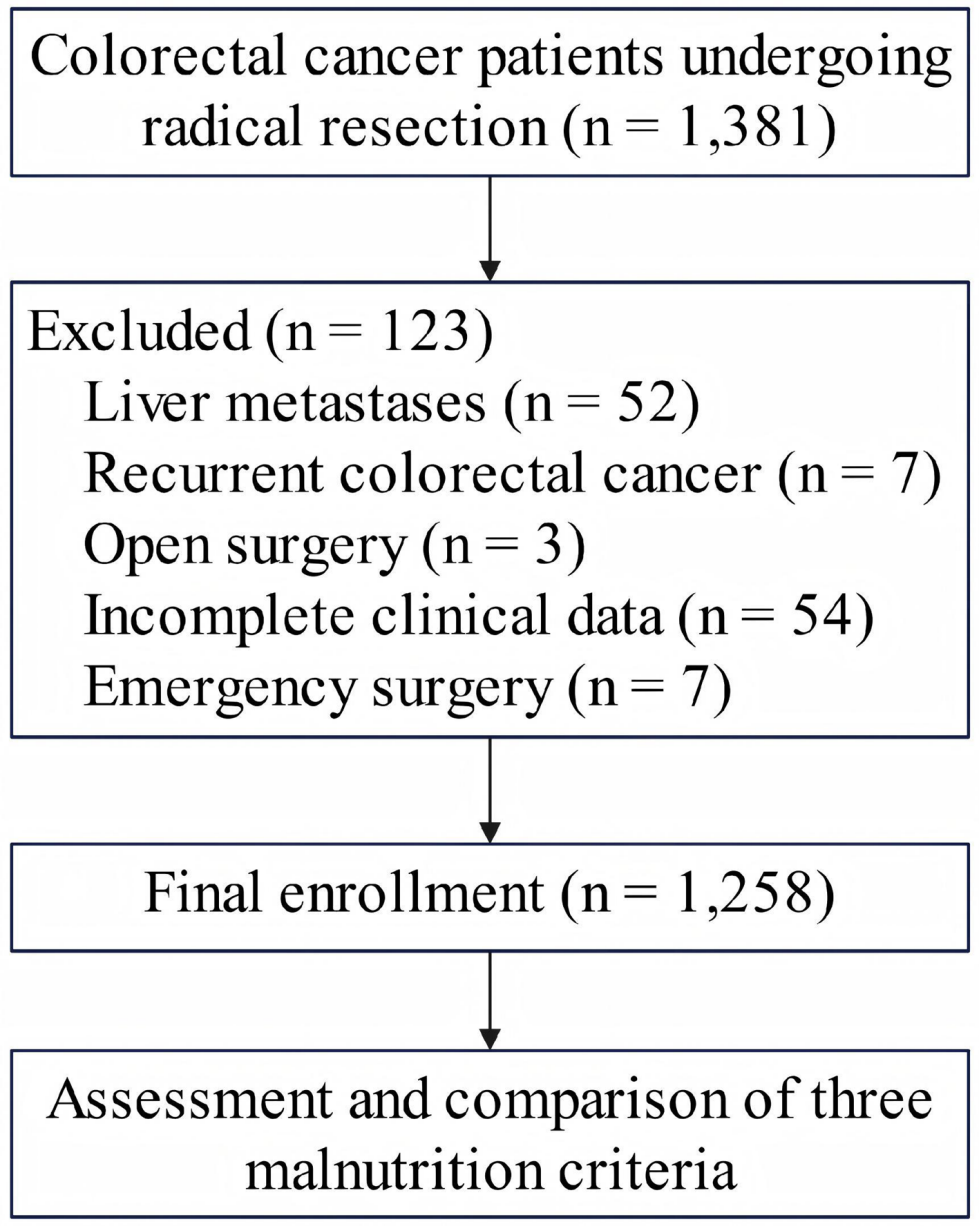
Flowchart of patient selection and enrollment.

### 2.2 Evaluation indicators

PG-SGA is a validated nutritional assessment tool specifically designed for patients with cancer. It comprises two components: a patient-reported section covering weight change, dietary intake, gastrointestinal symptoms, and functional capacity over the preceding month; and a clinician-rated section assessing disease status, metabolic demands, and physical examination findings (including muscle wasting, fat loss, and edema). Total scores range from 0 to 35, with higher scores indicating more severe malnutrition. PG-SGA evaluation is routinely performed at admission at our institution. In this study, a PG-SGA score ≥4 was defined as malnutrition (22, 23).

The ESPEN 2015 criteria diagnose malnutrition based on the presence of either of the following: (1) a body mass index (BMI) < 18.5 kg/m^2^; or (2) unintentional weight loss >10% over 6 months, or >5% over 3 months, in combination with either a reduced BMI (<20 kg/m^2^ in patients <70 years of age, or <22 kg/m^2^ in patients ≥70 years) or a reduced fat-free mass index (FFMI <17 kg/m^2^ in males and <15 kg/m^2^ in females) (24).

The GLIM criteria use a two-step diagnostic algorithm. Step 1 (Screening): all patients were screened using the Nutritional Risk Screening 2002 (NRS-2002), and those with a total score ≥3 were considered to be at nutritional risk and proceeded to GLIM diagnostic assessment, whereas those with a score <3 were classified as not meeting the GLIM criteria for malnutrition. Step 2 (Diagnosis): GLIM malnutrition was diagnosed when a patient fulfilled at least one phenotypic criterion and at least one etiologic criterion simultaneously. Phenotypic criteria include: (1) unintentional weight loss >5% within 6 months or >10% beyond 6 months; (2) low BMI (<18.5 kg/m^2^ in patients <70 years, or <20 kg/m^2^ in patients ≥70 years, in accordance with Asian-specific recommendations); and (3) reduced muscle mass, assessed using preoperative abdominal CT images. The L3 level was defined as the axial slice at the inferior endplate of the L3 vertebral body. Cross-sectional skeletal muscle area was quantified using Slice-O-Matic software with a semi-automated segmentation approach, and skeletal muscle was identified within a CT attenuation range of −29 to +150 Hounsfield units (HU). The muscles included in the analysis were the psoas, erector spinae, quadratus lumborum, transversus abdominis, internal oblique, external oblique, and rectus abdominis. SMI was calculated as skeletal muscle area (cm^2^) divided by height squared (m^2^), as illustrated in Supplementary Figure 1. Using established Asian-specific cut-offs, reduced muscle mass was defined as SMI < 40.8 cm^2^/m^2^ for males and <34.9 cm^2^/m^2^ for females (25–28). To assess measurement reliability, a random subset of 85 patients was independently analyzed by two trained observers, and one observer repeated the measurements after a 1-week interval. The intraobserver and interobserver intraclass correlation coefficients (ICCs) for SMI were 0.95 and 0.92, respectively, indicating excellent reproducibility. Since colorectal cancer is a malignancy characterized by chronic inflammation and disease burden, all enrolled patients were considered to fulfill the etiologic criterion for GLIM (29).

Postoperative complications were defined as Clavien-Dindo grade ≥ II events occurring within 30 days after surgery, including those during the index hospitalization, after discharge, or during readmission, identified through retrospective review of electronic medical records (30). Multiple complications in the same patient were counted separately. For all regression analyses, the outcome was the occurrence of at least one postoperative complication (yes vs. no). Respiratory disease was defined as a prior diagnosis of at least one of the following: asthma, chronic obstructive pulmonary disease, or restrictive lung disease (31). Overall survival (OS) was defined as the time interval from the date of surgery to death from any cause or the last follow-up date, which was April 2026. Preoperative anemia was defined as hemoglobin <120 g/L in males or <110 g/L in non-pregnant females (32). Preoperative hypoalbuminemia was defined as serum albumin <35 g/L (33).

Tumor location was categorized as follows: right-sided colon referred to malignancies located in the cecum, ascending colon, and hepatic flexure; left-sided colon referred to malignancies located in the splenic flexure, descending colon, sigmoid colon, and rectosigmoid junction; rectum referred to malignancies with the lower tumor margin within 12 cm of the anal verge (34).

The neutrophil-to-lymphocyte ratio (NLR) was calculated as the peripheral neutrophil count divided by the lymphocyte count, based on preoperative blood test results obtained within 1 week prior to surgery (35). The platelet-to-lymphocyte ratio (PLR) was calculated as the platelet count divided by the lymphocyte count using the same preoperative blood samples (36).

The variables included in the univariate and multivariable analyses were selected based on established clinical relevance and prior literature as potential risk factors for postoperative complications and overall survival in colorectal cancer. These included patient-related factors (e.g., age, diabetes, hypertension, respiratory disease, smoking, anemia, hypoalbuminemia, ASA grade), surgical factors (e.g., procedure, operative time, blood loss), and tumor-related factors (e.g., location, size, stage, differentiation, perineural and lymphovascular invasion).

### 2.3 Statistical analyses

Statistical analyses were performed using SPSS version 27.0 and R software version 4.5.2. Missing data were present for several variables, with missing rates ranging from 0.32 to 2.07% (Supplementary Table 1). For the primary (non-cross-validated) analyses, multiple imputation (MI) via chained equations was performed on the full dataset, and Rubin’s rules were used to pool regression coefficients and standard errors across imputed datasets. To avoid data leakage during internal cross-validation, MI was performed separately within each training fold rather than on the full dataset before cross-validation; the imputation model derived from each training fold was then applied to the corresponding held-out validation fold. Performance metrics were calculated within each imputed dataset and then averaged across imputed datasets and validation folds. Continuous variables were presented as mean ± standard deviation for normally-distributed data or as median [P25, P75] for non-normally-distributed data, and between-group comparisons were conducted using the independent-samples *t*-test or Mann–Whitney *U* test accordingly. Categorical variables were expressed as counts (percentages), and group differences were compared with the *χ*^2^ test.

Cohen’s Kappa coefficient was calculated to evaluate pairwise diagnostic agreement among the three malnutrition criteria. Kappa <0.4 indicated poor agreement, 0.4–0.6 indicated moderate agreement, and *κ* > 0.6 indicated substantial agreement. In addition, positive and negative agreement were calculated for each pairwise comparison (positive agreement = 2a/(2a + b + c); negative agreement = 2d/(2d + b + c)), where a and d denote concordant positive and negative results, respectively, and b and c denote discordant results in either direction.

Univariate analyses were first performed to screen risk variables associated with postoperative complications. Covariates were selected based on both univariable *p* < 0.05 and clinical relevance, as supported by previous literature and clinical knowledge. Three separate binary logistic regression models were then constructed. The dependent variable was the occurrence of postoperative complications (yes vs. no). The independent variables were one malnutrition definition (PG-SGA-defined, ESPEN-defined, or GLIM-defined malnutrition) together with an identical set of non-nutritional covariates across all three models. Model discrimination was assessed using the area under the ROC curve (AUC) based on the full cohort (apparent AUC), and pairwise comparisons were performed using DeLong’s test. To evaluate the robustness of the models and address potential optimism, internal validation was additionally performed using repeated 10-fold cross-validation with 5 repetitions (i.e., 50 training/validation splits). Within each training fold, univariable screening, variable selection, and multiple imputation were repeated, and the selected model was evaluated in the corresponding validation fold. The cross-validated AUC was calculated as the mean AUC across all validation folds from all repetitions and all imputed datasets. As an exploratory supplementary analysis, three independent random-forest classification models were built using the identical set of covariates. The dependent variable was postoperative complications, and the independent variables were the same set of covariates used in the logistic models. Mean Decrease Accuracy and Mean Decrease Gini were utilized to quantify variable importance. Random forest models were constructed using the random forest package in R with default parameters, except that variable importance was enabled to obtain Mean Decrease Accuracy and Mean Decrease Gini (Supplementary Table 2). Because feature-importance magnitudes across separate models cannot establish the comparative predictive performance of the three criteria, this analysis served as hypothesis-generating evidence only.

Univariate and multivariable Cox proportional-hazards regression models were applied for survival analysis. Covariates were selected based on both univariable *p* < 0.05 and clinical relevance. Three separate multivariable Cox models were established, including PG-SGA-defined malnutrition in Model 1, ESPEN-defined malnutrition in Model 2, and GLIM-defined malnutrition in Model 3. The dependent variables were overall survival time and status; the independent variables were one malnutrition definition and the same set of non-nutritional covariates across all three models. The proportional-hazards assumption was assessed using Schoenfeld residuals for all covariates included in the three multivariable Cox models, with both global and covariate-specific tests. A two-sided *p* < 0.05 was considered indicative of a violation. A stratified Cox model was prespecified as a sensitivity analysis in case of a violation. To compare the prognostic discriminative ability among the three malnutrition-incorporated models, Harrell’s C-index was calculated. Pairwise differences in the apparent C-index were evaluated using a bootstrap resampling procedure with 2,000 iterations; a 95% confidence interval not containing zero indicated statistical significance. In addition, internal validation was performed using repeated 10-fold cross-validation with 5 repetitions (i.e., 50 training/validation splits), with variable selection and fold-specific multiple imputation repeated within each training fold as described above. The cross-validated C-index was calculated as the mean C-index across all validation folds from all repetitions and all imputed datasets. Kaplan–Meier survival curves were plotted, and the log-rank test was used for inter-group survival comparisons.

To mitigate potential selection bias and balance baseline characteristics between patients with and without GLIM-defined malnutrition, a *post hoc* propensity-score matching (PSM) analysis was performed. Propensity scores were estimated using a multivariable logistic regression model that included a prespecified set of clinically relevant covariates: age, sex, preoperative anemia, preoperative hypoalbuminemia, NLR, PLR, and pathological stage. A 1:2 nearest-neighbor matching algorithm without replacement was used, with a caliper of 0.1 on the logit scale. Common support was assessed by visual inspection of propensity score distributions. Balance before and after matching was evaluated using standardized mean differences (SMDs), with an SMD < 0.1 considered indicative of adequate balance. All statistical tests were two-sided, and a significance level of *α* = 0.05 was adopted.

## 3 Results

### 3.1 Diagnostic consistency among three malnutrition assessment criteria

In the entire cohort, the prevalence of malnutrition as defined by PG-SGA, ESPEN, and GLIM criteria was 24.7% (311 patients), 15.7% (198 patients), and 29.9% (376 patients), respectively. PG-SGA and ESPEN agreed in 1,125 of 1,258 patients (89.4%), with 188 concordant positive and 937 concordant negative results; the kappa coefficient was 0.676 (*p* < 0.001), positive agreement was 73.9%, and negative agreement was 93.4%. ESPEN and GLIM agreed in 1,056 of 1,258 patients (83.9%), with 186 concordant positive and 870 concordant negative results; the kappa coefficient was 0.557 (*p* < 0.001), positive agreement was 64.8%, and negative agreement was 89.6%. PG-SGA and GLIM agreed in 989 of 1,258 patients (78.6%), with 209 concordant positive and 780 concordant negative results; the kappa coefficient was 0.463 (*p* < 0.001), positive agreement was 60.8%, and negative agreement was 85.3%. The cross-classification of diagnostic results for each pair of criteria is visualized in the confusion matrix plots (Figure 2).

**Figure 2:**
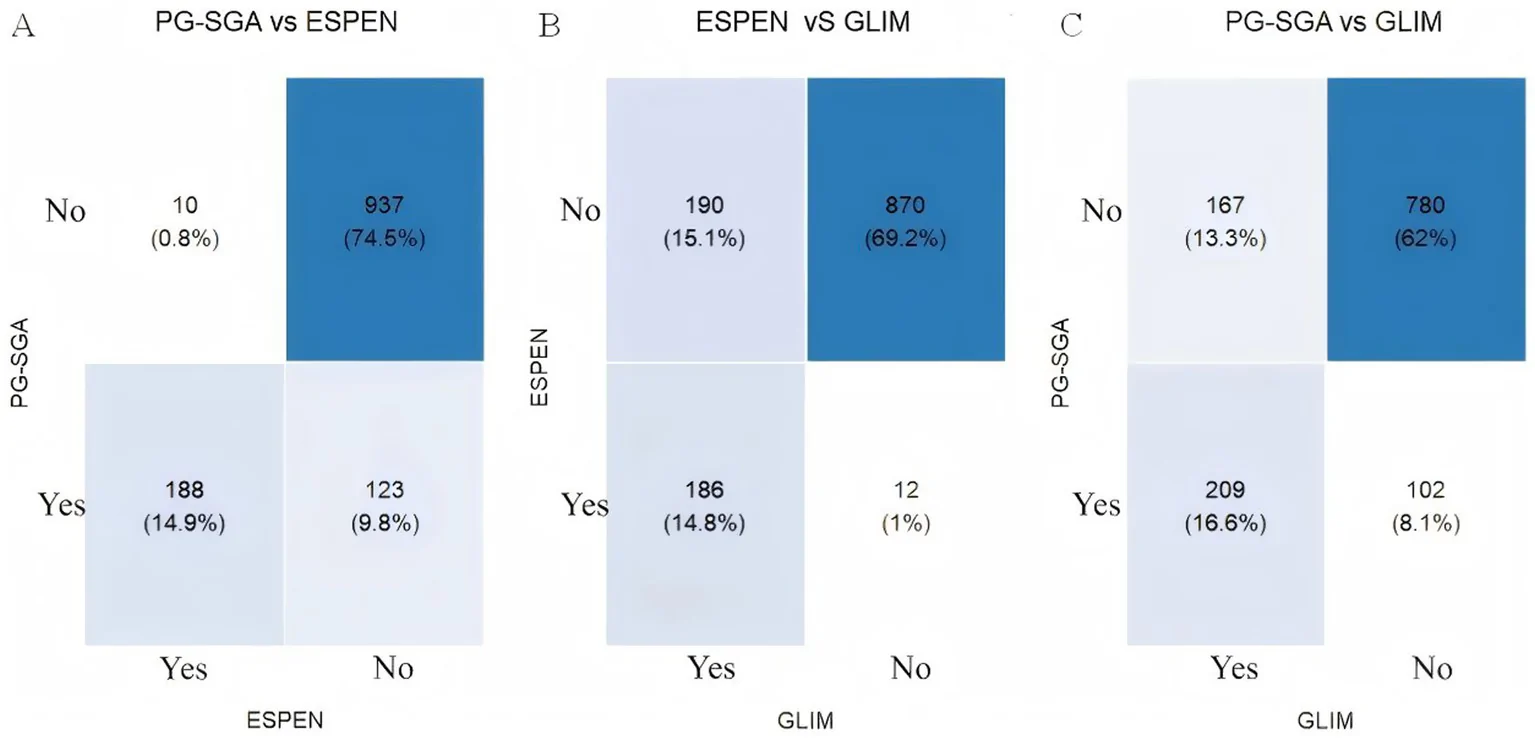
Confusion matrices for pairwise comparisons of the three malnutrition diagnostic criteria (PG-SGA, ESPEN, and GLIM) in patients with colorectal cancer. **(A)** PG-SGA vs. ESPEN; **(B)** ESPEN vs. GLIM; **(C)** PG-SGA vs. GLIM.

### 3.2 Univariate analysis of postoperative complications

Of the 1,258 patients with colorectal cancer included in this study, 187 (14.9%) developed postoperative complications. Pulmonary infection was the most frequent complication (Supplementary Table 3).

As shown in Table 1, univariate analysis revealed that age >65 years, diabetes mellitus, preoperative anemia, preoperative hypoalbuminemia, American Society of Anesthesiologists (ASA) grade III–IV, and operative time >3 h were significantly associated with an increased risk of postoperative complications (all *p* < 0.05). All three malnutrition criteria—PG-SGA-defined, ESPEN-defined, and GLIM-defined—were significantly associated with higher complication rates (*p* = 0.007, *p* = 0.037, and *p* < 0.001, respectively). No significant associations were observed for the remaining variables (all *p* > 0.05).

**Table 1:** Univariate analysis of postoperative complications in patients with colorectal cancer.

Characteristic	Complication group (*n* = 187)	No complication group (*n* = 1,071)	*p*-value
Sex			0.165
Male	121 (64.7)	639 (59.7)	
Female	66 (35.3)	432 (40.3)	
Age (years)			<0.001
≤65	98 (52.4)	706 (65.9)	
>65	89 (47.6)	365 (34.1)	
Hypertension			0.421
Yes	61 (32.6)	318 (29.7)	
No	126 (67.4)	753 (70.3)	
Diabetes mellitus			0.001
Yes	41 (21.9)	138 (12.9)	
No	146 (78.1)	933 (87.1)	
Respiratory disease			0.151
Yes	24 (12.8)	101 (9.4)	
No	163 (87.2)	970 (90.6)	
Smoking			0.113
Yes	39 (20.9)	173 (16.2)	
No	148 (79.1)	898 (83.8)	
Alcohol consumption			0.561
Yes	50 (26.7)	265 (24.7)	
No	137 (73.3)	806 (75.3)	
Previous abdominal surgery			0.240
Yes	24 (12.8)	107 (10.0)	
No	163 (87.2)	964 (90.0)	
Neoadjuvant therapy			0.438
Yes	59 (31.6)	308 (28.8)	
No	128 (68.4)	763 (71.2)	
Preoperative anemia			0.045
Yes	70 (37.4)	322 (30.1)	
No	117 (62.6)	749 (69.9)	
Preoperative hypoalbuminemia			0.037
Yes	45 (24.1)	189 (17.6)	
No	142 (75.9)	882 (82.4)	
Preoperative CEA (ng/mL)			0.261
≤5	108 (57.8)	665 (62.1)	
>5	79 (42.2)	406 (37.9)	
Preoperative CA19-9 (U/mL)			0.324
≤30	148 (79.1)	880 (82.2)	
>30	39 (20.9)	191 (17.8)	
Preoperative NLR	2.2 [1.6, 4.0]	2.2 [1.6, 3.0]	0.085
Preoperative PLR	153.5 [114.3, 208.6]	142.2 [106.2, 192.9]	0.067
ASA grade			0.004
I-II	145 (77.5)	918 (85.7)	
III-IV	42 (22.5)	153 (14.3)	
Surgical approach			0.109
Laparoscopic	164 (87.7)	889 (83.0)	
Robotic	23 (12.3)	182 (17.0)	
Operative time (h)			0.014
≤3	71 (38.0)	511 (47.7)	
>3	116 (62.0)	560 (52.3)	
Intraoperative blood loss (mL)			0.151
≤100	150 (80.2)	904 (84.4)	
>100	37 (19.8)	167 (15.6)	
Tumor location			0.094
Right colon	48 (25.7)	202 (18.9)	
Left colon	55 (29.4)	356 (33.2)	
Rectum	84 (44.9)	513 (47.9)	
Tumor size (cm)	4.3 ± 2.3	4.0 ± 1.8	0.103
Pathological stage			0.348
I	48 (25.7)	275 (25.7)	
II	70 (37.4)	348 (32.5)	
III	69 (36.9)	448 (41.8)	
Differentiation			0.635
Well/Moderately	156 (83.4)	908 (84.8)	
Poorly	31 (16.6)	163 (15.2)	
Perineural invasion			0.253
Yes	31 (16.6)	144 (13.4)	
No	156 (83.4)	927 (86.6)	
Lymphovascular invasion			0.401
Yes	27 (14.4)	131 (12.2)	
No	160 (85.6)	940 (87.8)	
PG-SGA-defined malnutrition			0.007
Yes	61 (32.6)	250 (23.3)	
No	126 (67.4)	821 (76.7)	
ESPEN-defined malnutrition			0.037
Yes	39 (20.9)	159 (14.8)	
No	148 (79.1)	912 (85.2)	
GLIM-defined malnutrition			<0.001
Yes	77 (41.2)	299 (27.9)	
No	110 (58.8)	772 (72.1)	

NLR, neutrophil-to-lymphocyte ratio; PLR, platelet-to-lymphocyte ratio; ASA, American Society of Anesthesiologists; CEA, carcinoembryonic antigen.

### 3.3 Multivariable logistic regression analysis of factors associated with postoperative complications

To adjust for potential confounders and evaluate the independent effects of the three malnutrition diagnostic criteria on postoperative complications, an identical set of non-nutritional covariates was selected based on both univariable significance and clinical relevance. These covariates (age, diabetes mellitus, preoperative anemia, preoperative hypoalbuminemia, ASA grade, and operative time) were entered together with one malnutrition indicator into three independent binary logistic regression models. Model 1 incorporated PG-SGA-defined malnutrition, Model 2 incorporated ESPEN-defined malnutrition, and Model 3 incorporated GLIM-defined malnutrition. The results of the multivariable analyses are presented as forest plots (Figures 3–5).

**Figure 3:**
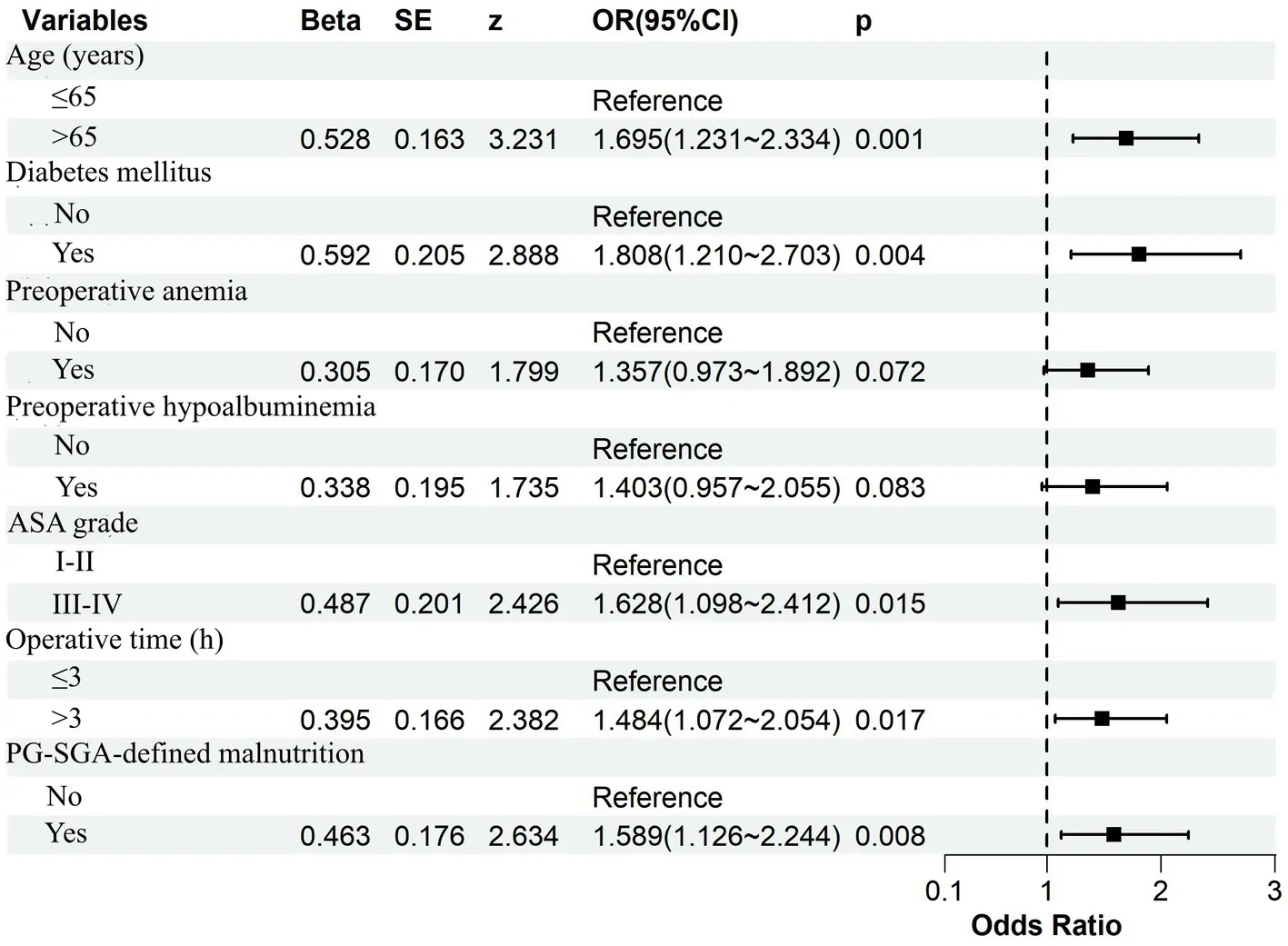
Forest plot of the multivariable logistic regression analysis for postoperative complications incorporating PG-SGA-defined malnutrition.

**Figure 4:**
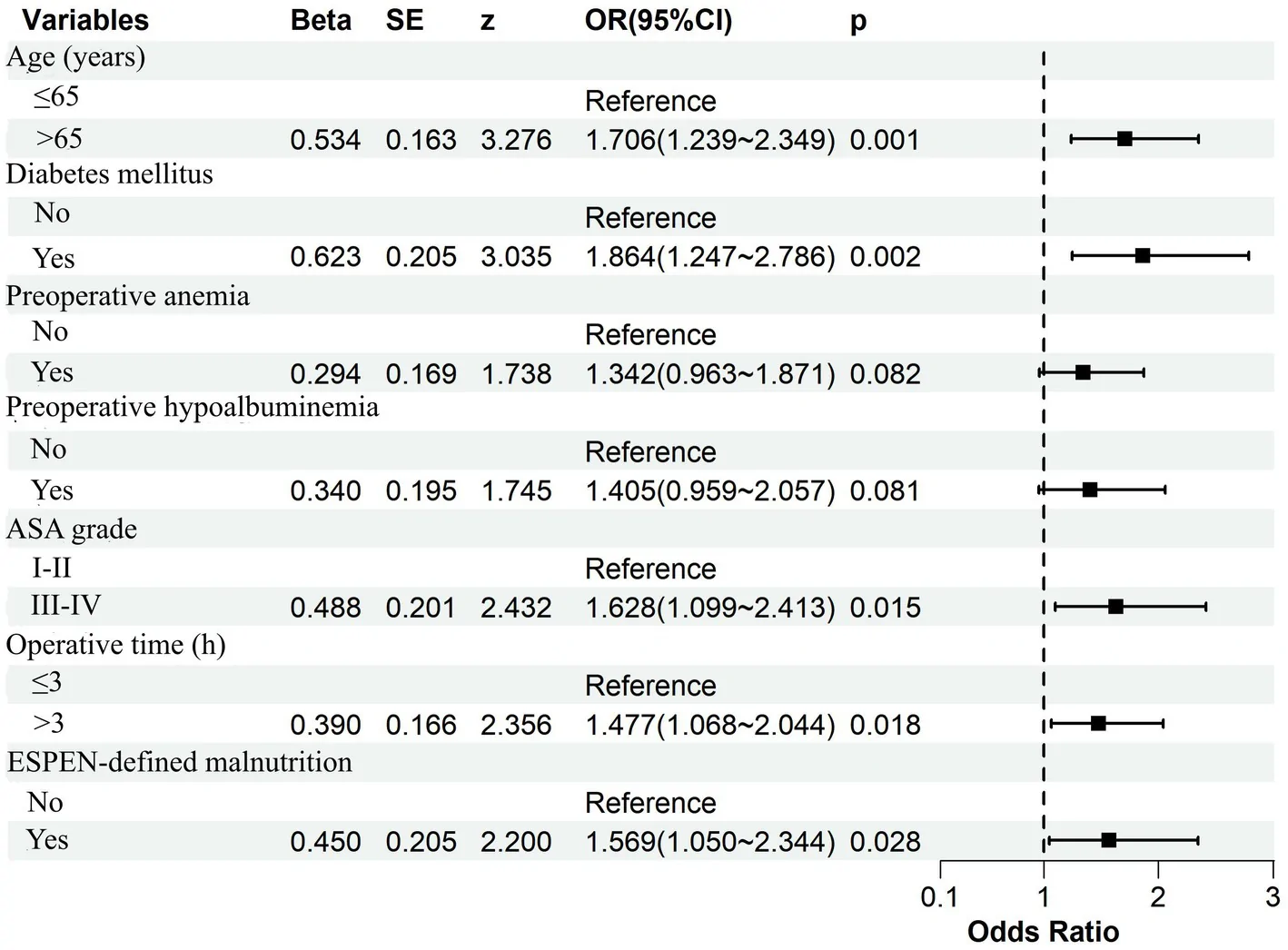
Forest plot of the multivariable logistic regression analysis for postoperative complications incorporating ESPEN-defined malnutrition.

**Figure 5:**
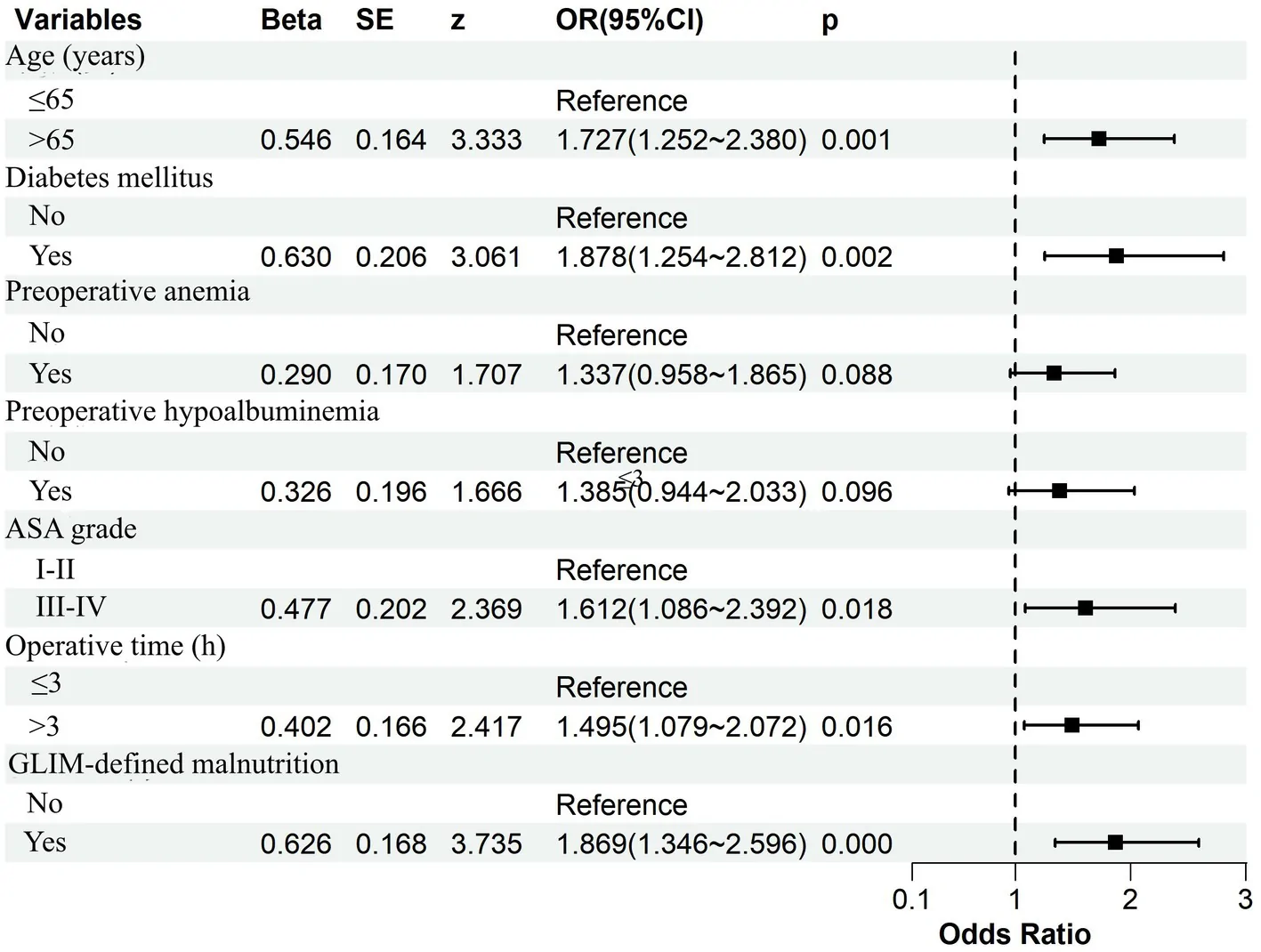
Forest plot of the multivariable logistic regression analysis for postoperative complications incorporating GLIM-defined malnutrition.

After adjusting for the aforementioned covariates, malnutrition defined by PG-SGA, ESPEN, and GLIM were all independent risk factors for postoperative complications in patients with colorectal cancer. Among the three criteria, GLIM-defined malnutrition yielded the highest odds ratio (OR = 1.869, 95% CI: 1.346–2.596, *p* < 0.001), followed by PG-SGA-defined malnutrition (OR = 1.589, 95% CI: 1.126–2.244, *p* = 0.008) and ESPEN-defined malnutrition (OR = 1.569, 95% CI: 1.050–2.344, *p* = 0.028). In addition, age >65 years, diabetes mellitus, ASA grade III–IV, and operative time >3 h remained independent risk factors for postoperative complications across all multivariable models (all *p* < 0.05).

The discriminative ability of each multivariable model for postoperative complications was further assessed by AUC analysis (Supplementary Figure 2). The apparent AUC values were 0.769 (95% CI: 0.728–0.809) for the PG-SGA-incorporated model, 0.764 (95% CI: 0.724–0.804) for the ESPEN-incorporated model, and 0.781 (95% CI: 0.743–0.820) for the GLIM-incorporated model. DeLong’s test for pairwise AUC comparisons revealed that only the GLIM-based model demonstrated a significantly higher AUC than the ESPEN-based model (*p* = 0.023), whereas no significant differences were detected between PG-SGA and ESPEN (*p* = 0.292) or between PG-SGA and GLIM (*p* = 0.172) (Supplementary Table 4). In the 10-fold cross-validation, the cross-validated AUCs were 0.767 for the PG-SGA model, 0.760 for the ESPEN model, and 0.776 for the GLIM model. These values were highly consistent with the apparent estimates, indicating minimal optimism and good internal validity.

### 3.4 Variable importance assessment of malnutrition indicators using random forest models

The random forest models, as an exploratory supplementary analysis, revealed that diabetes mellitus was consistently the most influential predictor of postoperative complications across all three models. Among the malnutrition indicators, GLIM-defined malnutrition demonstrated the highest importance values (Mean Decrease Accuracy: 10.9; Mean Decrease Gini: 5.3), followed by PG-SGA (10.2 and 4.6, respectively) and ESPEN (9.4 and 4.4, respectively). These exploratory findings were broadly consistent with the multivariable logistic regression results, but cross-model comparisons of feature importance should be interpreted with caution and do not establish superior predictive performance of GLIM (Supplementary Figures 3–5).

### 3.5 Univariate analysis of factors associated with overall survival

During a median follow-up of 80 months, 279 patients (22.2%) died, and 979 patients were censored, including 89 patients lost to follow-up; follow-up completeness was 92.9%. The 5-year overall survival rate was 76.1% (Supplementary Figure 6). The proportional-hazards assumption was satisfied for all covariates included in the three Cox models (global tests all *p* > 0.05; all covariate-specific *p* > 0.05). Univariate Cox proportional hazards regression analyses were performed to identify factors potentially influencing prognosis, with results detailed in Supplementary Table 5. Among clinicopathological variables, an age of >65 years, preoperative hypoalbuminemia, a preoperative CEA level of >5 ng/mL, right-sided colon tumor location, increased tumor size, advanced pathological stage, and poor differentiation were significantly associated with worse overall survival (all *p* < 0.05).

Regarding the three malnutrition assessment criteria, PG-SGA-defined malnutrition and GLIM-defined malnutrition were both significant risk factors for poor prognosis (*p* = 0.008 and *p* = 0.002, respectively), whereas ESPEN-defined malnutrition did not reach statistical significance (*p* = 0.091). Kaplan–Meier survival analyses corroborated these findings: as presented in Figure 6A–C, the 5-year survival rates for patients with versus without PG-SGA-defined malnutrition were 70.5 and 78.0%, respectively (*p* = 0.008); for ESPEN-defined malnutrition, the corresponding rates were 69.3 and 77.3% (*p* = 0.089); and for GLIM-defined malnutrition, the rates were 70.5 and 78.4% (*p* = 0.001).

**Figure 6:**
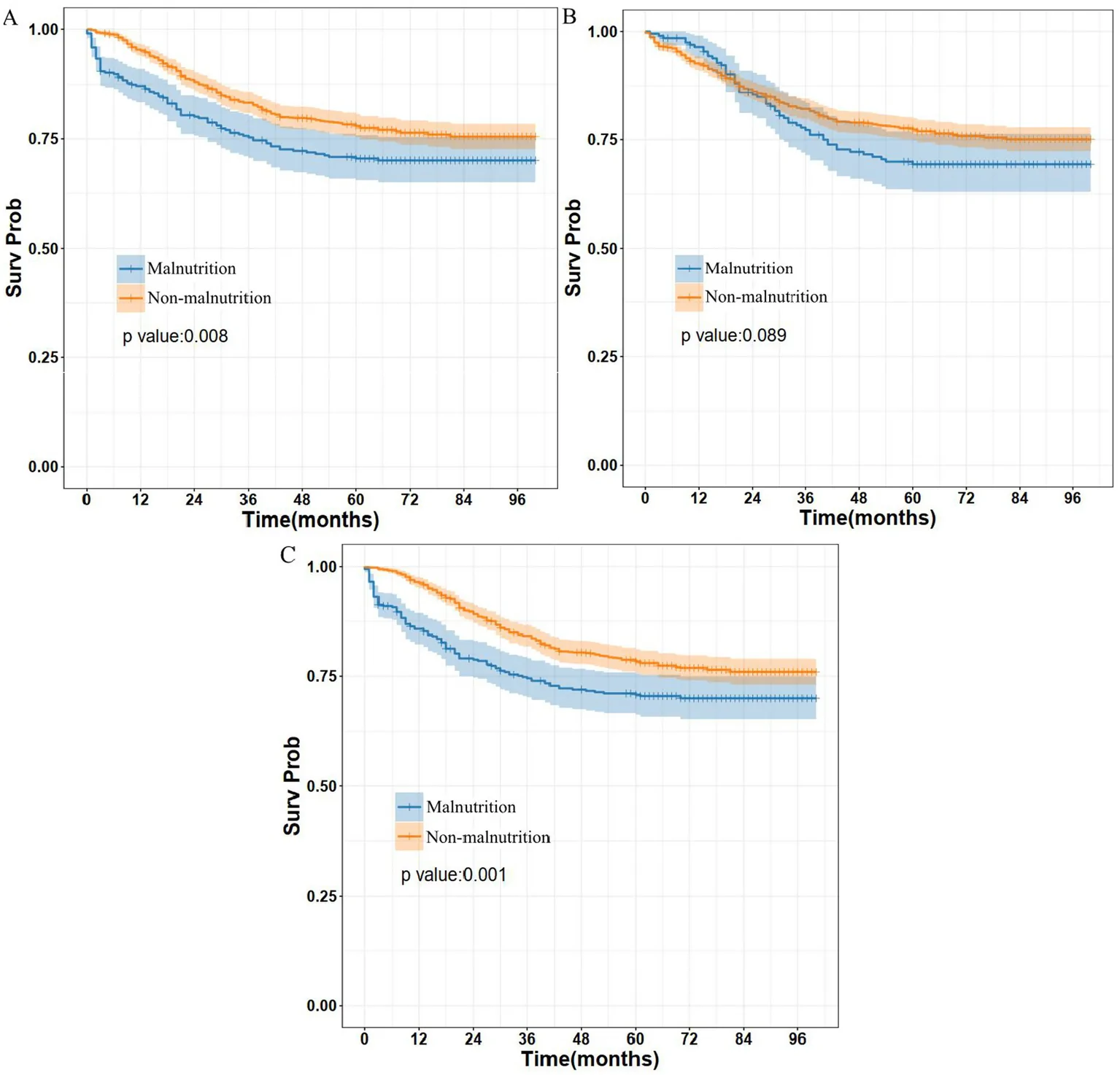
Kaplan–Meier survival curves comparing patients with and without malnutrition according to the three diagnostic criteria. **(A)** PG-SGA-defined malnutrition. **(B)** ESPEN-defined malnutrition. **(C)** GLIM-defined malnutrition.

### 3.6 Multivariable cox regression analysis of factors associated with overall survival

To further evaluate the independent prognostic value of the three malnutrition assessment criteria, an identical set of non-nutritional covariates was selected based on both univariable significance and clinical relevance. These covariates (age, preoperative hypoalbuminemia, preoperative CEA, tumor location, tumor size, pathological stage, and differentiation grade) were entered together with one malnutrition indicator into three separate multivariable Cox proportional hazards models: Model 1 incorporated PG-SGA-defined malnutrition, Model 2 incorporated ESPEN-defined malnutrition, and Model 3 incorporated GLIM-defined malnutrition. Although ESPEN-defined malnutrition did not reach statistical significance in the univariate analysis (*p* = 0.091), it was still included in the multivariable model for a comprehensive comparison.

As shown in Table 2, after adjusting for the aforementioned covariates, PG-SGA-defined malnutrition remained an independent risk factor for overall survival (HR = 1.364, 95% CI: 1.065–1.747, *p* = 0.014), whereas ESPEN-defined malnutrition was not independently associated with overall survival (HR = 1.251, 95% CI: 0.937–1.671, *p* = 0.129). In contrast, GLIM-defined malnutrition remained an independent prognostic factor for overall survival (HR = 1.415, 95% CI: 1.118–1.791, *p* = 0.004). Other independent prognostic factors, including age >65 years, preoperative CEA > 5 ng/mL, right-sided colon tumor location, and advanced pathological stage, were consistently observed across all models (all *p* < 0.05).

**Table 2:** Multivariable cox regression analysis for overall survival in patients with colorectal cancer.

Variable	Model 1 (PG-SGA)	*p*-value	Model 2 (ESPEN)	*p*-value	Model 3 (GLIM)	*p*-value
Age (years)						
≤65	Reference		Reference		Reference	
>65	1.425 (1.136–1.787)	0.002	1.429 (1.139–1.793)	0.002	1.439 (1.147–1.805)	0.002
Hypoalbuminemia						
No	Reference		Reference		Reference	
Yes	1.290 (0.982–1.696)	0.068	1.281 (0.974–1.684)	0.077	1.271 (0.966–1.671)	0.086
Preoperative CEA (ng/mL)						
≤5	Reference		Reference		Reference	
>5	1.351 (1.078–1.694)	0.009	1.344 (1.072–1.685)	0.010	1.335 (1.065–1.673)	0.012
Tumor location						
Right colon	Reference		Reference		Reference	
Left colon	0.641 (0.475–0.865)	0.004	0.636 (0.471–0.858)	0.003	0.641 (0.475–0.865)	0.004
Rectum	0.639 (0.484–0.843)	0.002	0.638 (0.483–0.842)	0.002	0.637 (0.482–0.840)	0.001
Tumor size (cm)	1.052 (0.994–1.113)	0.078	1.052 (0.994–1.112)	0.080	1.055 (0.997–1.116)	0.065
Pathological stage						
I	Reference		Reference		Reference	
II	1.636 (1.175–2.277)	0.004	1.673 (1.202–2.327)	0.002	1.662 (1.195–2.312)	0.003
III	2.067 (1.509–2.830)	<0.001	2.087 (1.524–2.857)	<0.001	2.079 (1.519–2.846)	<0.001
Differentiation						
Well/Moderately	Reference		Reference		Reference	
Poorly	1.294 (0.968–1.730)	0.082	1.288 (0.963–1.722)	0.088	1.261 (0.943–1.687)	0.117
Malnutrition						
No	Reference		Reference		Reference	
PG-SGA-defined malnutrition	1.364 (1.065–1.747)	0.014	-		-	-
ESPEN-defined malnutrition	-	-	1.251 (0.937–1.671)	0.129	-	-
GLIM-defined malnutrition	-	-	-	-	1.415 (1.118–1.791)	0.004

Three independent multivariable Cox regression models were fitted using identical sets of adjusting covariates. Model 1 incorporated PG-SGA-defined malnutrition; Model 2 incorporated ESPEN-defined malnutrition; Model 3 incorporated GLIM-defined malnutrition.

After adjusting for the aforementioned covariates, the apparent Harrell’s C-indexes of the PG-SGA-incorporated, ESPEN-incorporated, and GLIM-incorporated models were 0.730, 0.729, and 0.744, respectively (Supplementary Figure 7). The bootstrap-derived 95% CIs for the differences in C-index (GLIM minus PG-SGA and GLIM minus ESPEN) were 0.005 to 0.026 and 0.005 to 0.027, respectively. Since neither 95% CI contained zero, these findings indicated that the GLIM-based model provided a statistically significant improvement in prognostic discrimination over both the PG-SGA- and ESPEN-based models (both *p* < 0.05; Supplementary Table 6). In the 10-fold cross-validation, the cross-validated C-indexes were 0.734 for the PG-SGA model, 0.732 for the ESPEN model, and 0.747 for the GLIM model, which were highly consistent with the apparent estimates.

### 3.7 Comparison of baseline characteristics before and after PSM

Before matching, significant differences were observed between the two groups in age distribution, preoperative anemia, preoperative hypoalbuminemia, NLR, PLR, and pathological stage (all *p* < 0.05). After 1:2 nearest-neighbor matching without replacement, 933 patients were successfully matched, comprising 311 patients in the malnutrition group and 622 in the non-malnutrition group. Following matching, all baseline variables were well balanced between the two groups, with standardized mean differences <0.1 for all covariates, indicating comparable baseline characteristics (Supplementary Table 7 and Supplementary Figure 8).

### 3.8 Comparison of clinical outcomes between GLIM-defined malnutrition and non-malnutrition groups after PSM

For short-term outcomes, patients in the malnutrition group exhibited significantly longer postoperative hospital stay and a higher incidence of postoperative complications relative to the non-malnutrition group (both *p* < 0.001). No significant intergroup differences were observed in time to semi-liquid diet resumption, intraoperative or postoperative blood transfusion rate, in-hospital mortality, or 30-day readmission rate due to postoperative complications (Table 3, all *p* > 0.05).

**Table 3:** Comparison of short-term postoperative outcomes between GLIM-defined malnutrition and non-malnutrition groups after PSM.

Outcome	Malnutrition group (*n* = 311)	Non-malnutrition group (*n* = 622)	*p*-value
Time to semi-liquid diet (days)	3.9 ± 1.6	3.8 ± 2.1	0.302
Postoperative hospital stay (days)	8.4 ± 2.7	7.8 ± 2.1	<0.001
Postoperative complications			<0.001
Yes	73 (23.5)	81 (13.0)	
No	238 (76.5)	541 (87.0)	
Intraoperative or postoperative blood transfusion			0.413
Yes	8 (2.6)	11 (1.8)	
No	303 (97.4)	611 (98.2)	
In-hospital mortality			0.479
Yes	2 (0.6)	2 (0.3)	
No	309 (99.4)	620 (99.7)	
30-day readmission due to complications^*^			0.097
Yes	13 (4.2)	14 (2.3)	
No	298 (95.8)	608 (97.7)	

^*^Readmission due to postoperative complications within 30 days after discharge.

With respect to long-term survival, Kaplan–Meier analysis revealed that the 5-year overall survival rate was significantly lower in the GLIM-defined malnutrition group than in the non-malnutrition group (72.8% vs. 78.9%, *p* = 0.032; Figure 7).

**Figure 7:**
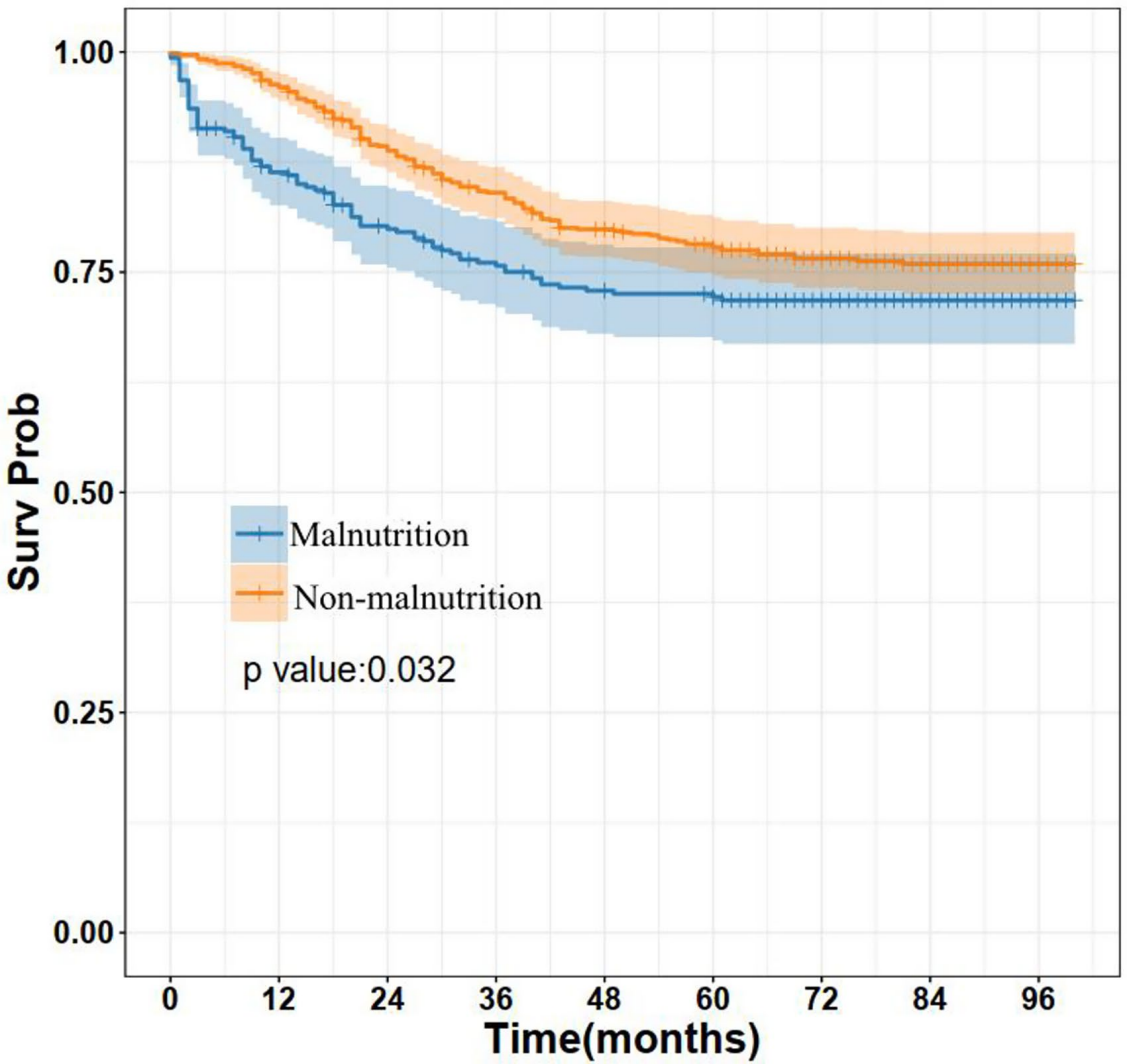
Kaplan–Meier survival curves comparing overall survival between the GLIM-defined malnutrition group and the non-malnutrition group after PSM.

## 4 Discussion

In this cohort, the prevalence of malnutrition defined by PG-SGA, ESPEN, and GLIM was 24.7%, 15.7%, and 29.9%, respectively, confirming that malnutrition is highly prevalent among patients undergoing radical resection for colorectal cancer. However, the reported prevalence of malnutrition in colorectal cancer patients varies considerably across studies. Using the GLIM criteria, Yan et al. (37) reported a malnutrition rate of 23.70% in a cohort of 983 patients who underwent radical resection for colorectal cancer. Shen et al. (38) applied the same criteria to 385 elderly patients undergoing colorectal cancer surgery and found a prevalence of 30.65%. In another study employing the PG-SGA in 266 patients with colorectal cancer, the prevalence was 27.0% (39). By contrast, a large-scale analysis of 4,487 patients reported an overall prevalence as high as 48.87% (40), whereas an analysis of the US National Inpatient Sample database (2011–2017) showed that only 11.05% of patients undergoing colorectal resection had documented malnutrition (11). These discrepancies likely stem from two main factors: the use of different assessment tools with varying diagnostic thresholds—particularly the lack of standardized methods and cut-offs for assessing reduced muscle mass—and the inherent heterogeneity of study populations in terms of geographic region, ethnicity, tumor stage, and socioeconomic status.

We further performed pairwise agreement analyses among the three criteria. The results showed substantial agreement between PG-SGA and ESPEN (*κ* = 0.676), and moderate agreement between ESPEN and GLIM (*κ* = 0.557) and between PG-SGA and GLIM (*κ* = 0.463), indicating that although the three criteria share moderate to substantial overlap in identifying malnutrition, they are not interchangeable. The differences in diagnostic frameworks and threshold definitions directly contribute to the gradient in malnutrition detection rates. Specifically, PG-SGA is a comprehensive tool specifically designed for cancer patients, encompassing multiple dimensions including weight change, dietary intake, gastrointestinal symptoms, functional status, and physical examination, with an emphasis on patient-reported subjective symptoms and functional impairment, thereby capturing a broader spectrum of nutritional risk (41). In contrast, the ESPEN criteria rely predominantly on objective anthropometric parameters such as unintentional weight loss and BMI, with relatively stringent diagnostic thresholds (15, 16, 42), which may explain the lowest detection rate observed in our cohort. The GLIM criteria adopt a two-step diagnostic algorithm, combining phenotypic criteria (weight loss, low BMI, and reduced muscle mass) with etiologic criteria following initial screening with NRS-2002. The inclusion of reduced muscle mass as a core phenotypic criterion enables GLIM to identify malnutrition in patients who may not be captured by weight loss or BMI alone, which likely accounts for the highest detection rate among the three tools.

Postoperative complications represent the most common short-term adverse events in colorectal cancer surgery, with significant clinical and economic implications (43, 44). Their occurrence not only prolongs hospital stay and increases healthcare costs, but may also delay or preclude adjuvant therapy, thereby negatively impacting long-term survival outcomes. In the present cohort, the postoperative complication rate was 14.9%, which is consistent with previously reported rates ranging from 10–25% in patients undergoing colorectal cancer surgery (45, 46).

In multivariable logistic regression analyses, all three malnutrition criteria were identified as independent risk factors for postoperative complications. Among them, GLIM-defined malnutrition yielded the highest odds ratio (OR = 1.869). To further compare the predictive discriminative ability of the three criteria beyond measures of association, we performed AUC comparisons using DeLong’s test. The GLIM-incorporated model achieved the highest AUC (0.781), with a modestly higher AUC than the ESPEN-based model (AUC = 0.764, *p* = 0.023), whereas no significant difference was observed between GLIM and PG-SGA (AUC = 0.769, *p* = 0.172). In an exploratory random forest analysis, GLIM-defined malnutrition showed the highest variable importance values, followed by PG-SGA and ESPEN. These rankings were broadly consistent with the AUC comparisons, but given the exploratory nature of this analysis, they should not be overinterpreted, and GLIM remained comparable to PG-SGA for postoperative complications.

Several mechanisms may explain the association between malnutrition and increased postoperative complications. First, protein deficiency in malnourished patients directly impairs collagen synthesis and tissue repair capacity, predisposing to anastomotic leakage and wound dehiscence (47). Second, malnutrition is frequently accompanied by immune dysfunction, characterized by reduced lymphocyte counts, decreased immunoglobulin synthesis, and diminished complement activity, which increases susceptibility to pathogens and elevates the risk of infectious complications, including surgical site infections, intra-abdominal infections, and pneumonia (48, 49). Third, preoperative malnutrition is often associated with skeletal muscle depletion and reduced physiological reserves, manifesting as impaired cardiopulmonary function, diminished respiratory muscle strength, and limited physical activity, all of which predispose patients to pulmonary complications, respiratory failure, and prolonged bedrest-related adverse events (50, 51).

In the present study, both PG-SGA- and GLIM-defined malnutrition were independent prognostic factors for overall survival (HR = 1.364, 95% CI: 1.065–1.747, *p* = 0.014 for PG-SGA; HR = 1.415, 95% CI: 1.118–1.791, *p* = 0.004 for GLIM), whereas ESPEN-defined malnutrition was not. To further compare the prognostic discriminative ability of the three criteria beyond the magnitude of association, we performed bootstrap-based pairwise comparisons of Harrell’s C-index. The GLIM-incorporated model demonstrated a statistically significant but modest improvement in C-index over both the PG-SGA-incorporated model (ΔC-index = 0.0145, 95% CI: 0.0054–0.0261) and the ESPEN-incorporated model (ΔC-index = 0.0150, 95% CI: 0.0054–0.0271), both *p* < 0.05. These findings indicate that, independently of traditional prognostic factors, GLIM provides modestly better prognostic discrimination for long-term survival; however, the absolute improvement (ΔC-index ≈ 0.015) was small, and statistical significance should be distinguished from clinical relevance, which remains to be established.

The modestly better prognostic performance of GLIM may be attributed to its inclusion of reduced muscle mass as a core phenotypic criterion. Sarcopenia, defined as low muscle mass and function, has been consistently associated with poor prognosis in colorectal cancer patients, independently of BMI (52–55). It reflects not only nutritional depletion but also the systemic catabolic state driven by tumor-related chronic inflammation and hypermetabolism. Patients with reduced muscle mass are often characterized by diminished physiological reserves, poorer tolerance to treatment, and a higher likelihood of disease progression, all of which contribute to adverse survival outcomes (56–58). In contrast, the ESPEN criteria rely heavily on BMI and weight loss—parameters that may fail to capture the “hidden” malnutrition in patients who are overweight or obese but have significant muscle depletion, a condition known as sarcopenic obesity. This limitation may explain why ESPEN-defined malnutrition failed to predict OS in our cohort.

The mechanisms linking malnutrition to worse survival in colorectal cancer patients are multifactorial. First, malnutrition impairs protein synthesis and immune function, compromising the body’s ability to mount effective immune surveillance against minimal residual disease and circulating tumor cells, thereby increasing the risk of postoperative recurrence and metastasis (59). Second, malnutrition is frequently accompanied by a systemic inflammatory response. Proinflammatory cytokines accelerate skeletal muscle proteolysis, creating a vicious cycle of “inflammation-driven depletion” that promotes tumor progression and is closely associated with chemoresistance (60, 61). Third, malnourished patients often have poor performance status, which may lead to treatment delays, dose reductions, or premature discontinuation of adjuvant therapy, ultimately compromising long-term outcomes (62, 63).

To further explore the association between GLIM-defined malnutrition and clinical outcomes, a *post hoc* PSM analysis was performed for the GLIM criterion, as it was the criterion used in the primary analyses that showed the most favorable predictive profile among the three. Before matching, significant differences were observed between the GLIM-defined malnutrition and non-malnutrition groups in age distribution, preoperative anemia, preoperative hypoalbuminemia, NLR, PLR, and pathological stage. These baseline imbalances are biologically plausible: older patients are more susceptible to nutritional depletion due to age-related physiological decline and comorbidities (64); anemia and hypoalbuminemia are direct manifestations of poor nutritional status and systemic inflammation (65, 66); elevated NLR and PLR reflect the chronic inflammatory state commonly seen in malnourished cancer patients (67); and advanced pathological stage is associated with greater tumor burden and metabolic stress, which predispose to malnutrition (68). After 1:2 nearest-neighbor matching, all baseline covariates were well balanced between the two groups, with standardized mean differences <0.1 for all covariates. In the matched cohort, patients with GLIM-defined malnutrition continued to exhibit significantly prolonged postoperative hospital stay, higher rates of postoperative complications, and worse long-term overall survival. These findings suggest that GLIM-defined malnutrition is associated with adverse clinical outcomes in patients with colorectal cancer; however, causality cannot be established in this retrospective observational dataset.

Several limitations of this study should be acknowledged. First, this was a single-center retrospective observational study. Although PG-SGA assessment was routinely performed in all hospitalized patients, the ESPEN and GLIM criteria were applied retrospectively based on documented anthropometric and laboratory data, which precludes us from completely ruling out inherent selection bias and residual confounding from unmeasured factors. Second, the study population consisted exclusively of Asian patients. Although the SMI cut-offs used to define reduced muscle mass were derived from Asian-specific reference data, the lack of standardized cut-offs across different populations remains a concern. Third, the exclusion of emergency and open surgery (7 and 3 patients, respectively) may limit generalizability to frailer patients with higher malnutrition and complication rates. Fourth, we only evaluated all-cause overall survival and did not analyze recurrence-free survival or disease-specific survival as endpoints, which may provide a more comprehensive picture of oncological outcomes. Finally, the PSM analysis was *post hoc*; it can reduce measured confounding but cannot establish causality in this retrospective observational dataset; in addition, assuming all patients fulfilled the GLIM etiologic criterion based on cancer diagnosis alone may have overestimated malnutrition prevalence.

This study demonstrates that PG-SGA, ESPEN, and GLIM criteria are not interchangeable for identifying malnutrition in patients undergoing curative resection for colorectal cancer. For postoperative complications, GLIM showed better discriminative ability than ESPEN but was comparable to PG-SGA. For long-term overall survival, GLIM provided modestly better prognostic discrimination compared with both PG-SGA and ESPEN, although the absolute improvement was small. These findings suggest that GLIM may serve as a complementary tool for perioperative nutritional risk assessment in colorectal cancer surgery; however, the clinical importance of these relatively small performance differences warrants further validation.

## Data Availability

The original contributions presented in the study are included in the article/Supplementary material, further inquiries can be directed to the corresponding author/s.
