# Supplementary material for: Comparison of three malnutrition assessment criteria for predicting postoperative complications and overall survival in colorectal cancer
Source: Front Nutr. 2026 Oct 28;13:1996149. doi: 10.3389/fnut.2026.1996149 (PMC13752205; doi:10.3389/fnut.2026.1996149)
Supplement: Supplementary file 1 [file Supplementary_file_1.doc]

**Supplementary Material**

**SUPPLEMENTARY FIGURE 1**

Example of skeletal muscle area measurement at the L3 vertebral level on axial computed tomography.

**SUPPLEMENTARY TABLE 1 Missing data for each variable in the cohort (n = 1,258).**

| Variable | Missing (n) | Missing (%) |
| --- | --- | --- |
| Sex | 0 | 0 |
| Age (years) | 0 | 0 |
| Hypertension | 18 | 1.43 |
| Diabetes mellitus | 19 | 1.51 |
| Respiratory disease | 26 | 2.07 |
| Smoking | 19 | 1.51 |
| Alcohol consumption | 15 | 1.19 |
| Previous abdominal surgery | 17 | 1.35 |
| Neoadjuvant therapy | 0 | 0 |
| Preoperative anemia | 0 | 0 |
| Preoperative hypoalbuminemia | 0 | 0 |
| Preoperative CEA (ng/mL) | 7 | 0.56 |
| Preoperative CA19‑9 (U/mL) | 4 | 0.32 |
| Preoperative NLR | 0 | 0 |
| Preoperative PLR | 0 | 0 |
| ASA grade | 0 | 0 |
| Surgical approach | 0 | 0 |
| Operative time (h) | 0 | 0 |
| Intraoperative blood loss (mL) | 0 | 0 |
| Tumor location | 0 | 0 |
| Tumor size (cm) | 0 | 0 |
| Pathological stage | 0 | 0 |
| Differentiation | 0 | 0 |
| Perineural invasion | 0 | 0 |
| Lymphovascular invasion | 0 | 0 |

**SUPPLEMENTARY TABLE 2** Default parameters for the random forest models.

| Parameter | Value |
| --- | --- |
| Package | randomForest (R version 4.5.2) |
| Number of trees (ntree) | 500 |
| mtry | floor(sqrt(p)), where p = number of predictors |
| Node size (nodesize) | 1 |
| Max nodes (maxnodes) | NULL |
| Sampling | with replacement (replace = TRUE) |
| Sample size (sampsize) | nrow(x) |
| Variable importance | enabled (importance = TRUE) |
| Other parameters | default |

**SUPPLEMENTARY TABLE 3 Postoperative complications in the entire cohort (n = 1,258).**

| Complication | No. of cases | Percentage (%) |
| --- | --- | --- |
| Pulmonary infection | 117 | 9.3 |
| Wound infection | 15 | 1.2 |
| Intestinal obstruction | 19 | 1.5 |
| Anastomotic leakage | 26 | 2.1 |
| Bleeding | 15 | 1.2 |
| Venous thrombosis | 4 | 0.3 |
| Pleural effusion | 47 | 3.7 |
| Intra-abdominal abscess | 18 | 1.4 |

Multiple complications per patient were counted separately; percentages use the entire cohort (n = 1,258) as the denominator.

**SUPPLEMENTARY FIGURE 2**

ROC curves of three multivariable models for predicting postoperative complications. (**A**) Model incorporating PG‑SGA‑defined malnutrition; (**B**) Model incorporating ESPEN‑defined malnutrition; (**C**) Model incorporating GLIM‑defined malnutrition.

**SUPPLEMENTARY TABLE 4 Pairwise comparisons of AUCs for predicting postoperative complications using DeLong’s test.**

| Comparison | Z‑statistic | P value |
| --- | --- | --- |
| PG‑SGA vs. ESPEN | 1.054 | 0.292 |
| PG‑SGA vs. GLIM | 1.366 | 0.172 |
| ESPEN vs. GLIM | 2.278 | 0.023 |

**SUPPLEMENTARY FIGURE 3**

Variable importance plots from the random forest model incorporating PG-SGA-defined malnutrition for predicting postoperative complications. (**A**) Mean Decrease Accuracy; (**B**) Mean Decrease Gini.

**SUPPLEMENTARY FIGURE 4**

Variable importance plots from the random forest model incorporating ESPEN-defined malnutrition for predicting postoperative complications. (**A**) Mean Decrease Accuracy; (**B**) Mean Decrease Gini.

**SUPPLEMENTARY FIGURE 5**

Variable importance plots from the random forest model incorporating GLIM-defined malnutrition for predicting postoperative complications. (**A**) Mean Decrease Accuracy; (**B**) Mean Decrease Gini.

**SUPPLEMENTARY FIGURE 6**

Kaplan-Meier curve of overall survival for the entire cohort of patients with colorectal cancer.

**SUPPLEMENTARY TABLE 5 Univariate cox regression analysis of factors associated with overall survival in patients with colorectal cancer.**

| Characteristic | HR (95% CI) | P value |
| --- | --- | --- |
| Sex |  |  |
| Male | Reference |  |
| Female | 0.815(0.646–1.029) | 0.085 |
| Age (years) |  |  |
| ≤65 | Reference |  |
| >65 | 1.433(1.144–1.795) | 0.002 |
| Hypertension |  |  |
| No | Reference |  |
| Yes | 1.086(0.854–1.382) | 0.499 |
| Diabetes mellitus |  |  |
| No | Reference |  |
| Yes | 1.167(0.860–1.584) | 0.321 |
| Respiratory disease |  |  |
| No | Reference |  |
| Yes | 1.227(0.863–1.745) | 0.254 |
| Smoking |  |  |
| No | Reference |  |
| Yes | 1.161(0.867–1.554) | 0.317 |
| Alcohol consumption |  |  |
| No | Reference |  |
| Yes | 0.877(0.671–1.144) | 0.333 |
| Previous abdominal surgery |  |  |
| No | Reference |  |
| Yes | 1.268(0.903–1.781) | 0.171 |
| Preoperative anemia |  |  |
| No | Reference |  |
| Yes | 1.107(0.873–1.404) | 0.402 |
| Preoperative hypoalbuminemia |  |  |
| No | Reference |  |
| Yes | 1.326(1.010–1.741) | 0.042 |
| Preoperative CEA (ng/mL) |  |  |
| ≤5 | Reference |  |
| >5 | 1.310(1.046–1.640) | 0.019 |
| Preoperative CA19-9 (U/mL) |  |  |
| ≤30 | Reference |  |
| >30 | 1.220(0.927–1.607) | 0.156 |
| Preoperative NLR | 1.016(0.998–1.034) | 0.076 |
| Preoperative PLR | 1.001(1.000–1.002) | 0.109 |
| ASA grade |  |  |
| I-II | Reference |  |
| III-IV | 1.156(0.860–1.554) | 0.337 |
| Surgical approach |  |  |
| Laparoscopic | Reference |  |
| Robotic | 0.878(0.644–1.198) | 0.412 |
| Operative time(h) |  |  |
| ≤3 | Reference |  |
| >3 | 1.180(0.941–1.479) | 0.152 |
| Intraoperative blood loss (mL) |  |  |
| ≤100 | Reference |  |
| >100 | 1.140(0.853–1.523) | 0.375 |
| Tumor location |  |  |
| Right colon | Reference |  |
| Left colon | 0.617(0.458–0.832) | 0.002 |
| Rectum | 0.623(0.473–0.822) | < 0.001 |
| Tumor size (cm) | 1.061(1.003–1.122) | 0.039 |
| Pathological stage |  |  |
| I | Reference |  |
| II | 1.684(1.211–2.342) | 0.002 |
| III | 2.089(1.527–2.859) | < 0.001 |
| Differentiation |  |  |
| Well/Moderately | Reference |  |
| Poorly | 1.363(1.020–1.821) | 0.036 |
| Perineural invasion |  |  |
| No | Reference |  |
| Yes | 1.243(0.916–1.687) | 0.163 |
| Lymphovascular invasion |  |  |
| No | Reference |  |
| Yes | 1.317(0.966–1.797) | 0.082 |
| Postoperative complications |  |  |
| No | Reference |  |
| Yes | 1.029(0.745–1.420) | 0.864 |
| PG-SGA-defined malnutrition |  |  |
| No | Reference |  |
| Yes | 1.394(1.091–1.782) | 0.008 |
| ESPEN-defined malnutrition |  |  |
| No | Reference |  |
| Yes | 1.282(0.961–1.709) | 0.091 |
| GLIM-defined malnutrition |  |  |
| No | Reference |  |
| Yes | 1.457(1.153–1.841) | 0.002 |

NLR: neutrophil‑to‑lymphocyte ratio; PLR: platelet‑to‑lymphocyte ratio; ASA: American Society of Anesthesiologists; CEA: carcinoembryonic antigen.

**SUPPLEMENTARY FIGURE 7**

Time‑dependent C‑index curves of the multivariable Cox models incorporating different malnutrition criteria for predicting overall survival in patients with colorectal cancer. (**A**) Model incorporating PG-SGA-defined malnutrition; (**B**) Model incorporating ESPEN-defined malnutrition; (**C**) Model incorporating GLIM-defined malnutrition.

**SUPPLEMENTARY TABLE 6 Bootstrap comparison of C-index between GLIM versus PG-SGA/ESPEN models for overall survival.**

| Pairwise Comparison | ΔC‑index (95% CI) | P value |
| --- | --- | --- |
| GLIM vs PG-SGA | 0.015 (0.005 to 0.026) | < 0.05 |
| GLIM vs ESPEN | 0.015 (0.005 to 0.027) | < 0.05 |

**SUPPLEMENTARY TABLE 7 Comparison of baseline characteristics between GLIM-defined malnutrition and non‑malnutrition groups before and after PSM.**

| Characteristic | Before matching | | |  |  | After matching | | |  |
| --- | --- | --- | --- | --- | --- | --- | --- | --- | --- |
|  | Malnutrition (n = 376) | Non-malnutrition (n = 882) | P value | SMD |  | Malnutrition (n = 311) | Non-malnutrition (n = 622) | P value | SMD |
| Sex |  |  | 0.125 | 0.093 |  |  |  | 0.756 | 0.027 |
| Male | 240 (63.8) | 520 (59.0) |  |  |  | 196 (63.0) | 384 (61.7) |  |  |
| Female | 136 (36.2) | 362 (41.0) |  |  |  | 115 (37.0) | 238 (38.3) |  |  |
| Age, years |  |  | 0.043 | 0.128 |  |  |  | 0.547 | 0.047 |
| ≤ 65 | 224 (59.6) | 580 (65.8) |  |  |  | 203 (65.3) | 392 (63.0) |  |  |
| > 65 | 152 (40.4) | 302 (34.2) |  |  |  | 108 (34.7) | 230 (37.0) |  |  |
| Hypertension |  |  | 0.363 | 0.061 |  |  |  | 0.741 | 0.028 |
| Yes | 106 (28.2) | 273 (31.0) |  |  |  | 88 (28.3) | 184 (29.6) |  |  |
| No | 270 (71.8) | 609 (69.0) |  |  |  | 223 (71.7) | 438 (70.4) |  |  |
| Diabetes mellitus |  |  | 0.290 | 0.072 |  |  |  | 0.428 | 0.063 |
| Yes | 47 (12.5) | 132 (15.0) |  |  |  | 36 (11.6) | 85 (13.7) |  |  |
| No | 329 (87.5) | 750 (85.0) |  |  |  | 275 (88.4) | 537 (86.3) |  |  |
| Respiratory disease |  |  | 0.660 | 0.033 |  |  |  | 0.728 | 0.032 |
| Yes | 40 (10.6) | 85 ( 9.6) |  |  |  | 29 ( 9.3) | 64 (10.3) |  |  |
| No | 336 (89.4) | 797 (90.4) |  |  |  | 282 (90.7) | 558 (89.7) |  |  |
| Smoking |  |  | 0.181 | 0.086 |  |  |  | 0.975 | 0.009 |
| Yes | 72 (19.1) | 140 (15.9) |  |  |  | 52 (16.7) | 102 (16.4) |  |  |
| No | 304 (80.9) | 742 (84.1) |  |  |  | 259 (83.3) | 520 (83.6) |  |  |
| Alcohol consumption |  |  | 0.738 | 0.025 |  |  |  | 0.535 | 0.048 |
| Yes | 97 (25.8) | 218 (24.7) |  |  |  | 80 (25.7) | 147 (23.6) |  |  |
| No | 279 (74.2) | 664 (75.3) |  |  |  | 231 (74.3) | 475 (76.4) |  |  |
| Previous abdominal surgery |  |  | 0.281 | 0.071 |  |  |  | 0.909 | 0.016 |
| Yes | 45 (12.0) | 86 ( 9.8) |  |  |  | 31 (10.0) | 65 (10.5) |  |  |
| No | 331 (88.0) | 796 (90.2) |  |  |  | 280 (90.0) | 557 (89.5) |  |  |
| Neoadjuvant therapy |  |  | 0.184 | 0.085 |  |  |  | 0.739 | 0.028 |
| Yes | 120 (31.9) | 247 (28.0) |  |  |  | 91 (29.3) | 174 (28.0) |  |  |
| No | 256 (68.1) | 635 (72.0) |  |  |  | 220 (70.7) | 448 (72.0) |  |  |
| Preoperative anemia |  |  | 0.030 | 0.137 |  |  |  | 0.744 | 0.028 |
| Yes | 134 (35.6) | 258 (29.3) |  |  |  | 98 (31.5) | 188 (30.2) |  |  |
| No | 242 (64.4) | 624 (70.7) |  |  |  | 213 (68.5) | 434 (69.8) |  |  |
| Preoperative hypoalbuminemia |  |  | 0.002 | 0.191 |  |  |  | 0.976 | 0.008 |
| Yes | 90 (23.9) | 144 (16.3) |  |  |  | 54 (17.4) | 110 (17.7) |  |  |
| No | 286 (76.1) | 738 (83.7) |  |  |  | 257 (82.6) | 512 (82.3) |  |  |
| Preoperative CEA, ng/mL |  |  | 0.113 | 0.095 |  |  |  | 0.757 | 0.027 |
| ≤ 5 | 218 (58.0) | 555 (62.9) |  |  |  | 195 (62.7) | 382 (61.4) |  |  |
| > 5 | 158 (42.0) | 327 (37.1) |  |  |  | 116 (37.3) | 240 (38.6) |  |  |
| Preoperative CA19-9, U/mL |  |  | 0.282 | 0.070 |  |  |  | 0.835 | 0.021 |
| ≤ 30 | 300 (79.8) | 728 (82.5) |  |  |  | 255 (82.0) | 505 (81.2) |  |  |
| > 30 | 76 (20.2) | 154 (17.5) |  |  |  | 56 (18.0) | 117 (18.8) |  |  |
| Preoperative NLR | 2.3 [1.7, 3.4] | 2.1 [1.6, 3.0] | 0.048 | 0.110 |  | 2.2 [1.6, 3.2] | 2.1 [1.6, 3.1] | 0.667 | 0.029 |
| Preoperative PLR | 150.8 [111.1, 203.3] | 140.0 [105.3, 194.6] | 0.002 | 0.182 |  | 148.5 [108.3, 187.7] | 148.2 [108.4, 201.8] | 0.649 | 0.032 |
| ASA grade |  |  | 0.290 | 0.070 |  |  |  | 0.730 | 0.030 |
| I-II | 311 (82.7) | 752 (85.3) |  |  |  | 258 (83.0) | 523 (84.1) |  |  |
| III-IV | 65 (17.3) | 130 (14.7) |  |  |  | 53 (17.0) | 99 (15.9) |  |  |
| Surgical approach |  |  | 0.426 | 0.055 |  |  |  | 0.515 | 0.052 |
| Laparoscopic | 320 (85.1) | 733 (83.1) |  |  |  | 263 (84.6) | 514 (82.6) |  |  |
| Robotic | 56 (14.9) | 149 (16.9) |  |  |  | 48 (15.4) | 108 (17.4) |  |  |
| Operative time, h |  |  | 0.238 | 0.076 |  |  |  | 0.532 | 0.048 |
| ≤3 | 184 (48.9) | 398 (45.1) |  |  |  | 144 (46.3) | 303 (48.7) |  |  |
| > 3 | 192 (51.1) | 484 (54.9) |  |  |  | 167 (53.7) | 319 (51.3) |  |  |
| Intraoperative blood loss, mL |  |  | 0.806 | 0.020 |  |  |  | 0.773 | 0.027 |
| ≤100 | 317 (84.3) | 737 (83.6) |  |  |  | 261 (83.9) | 528 (84.9) |  |  |
| > 100 | 59 (15.7) | 145 (16.4) |  |  |  | 50 (16.1) | 94 (15.1) |  |  |
| Tumor location |  |  | 0.190 | 0.091 |  |  |  | 0.548 | 0.076 |
| Right-sided colon | 78 (20.7) | 172 (19.5) |  |  |  | 68 (21.9) | 119 (19.1) |  |  |
| Left-sided colon | 109 (29.0) | 302 (34.2) |  |  |  | 94 (30.2) | 204 (32.8) |  |  |
| Rectum | 189 (50.3) | 408 (46.3) |  |  |  | 149 (47.9) | 299 (48.1) |  |  |
| Tumor size, cm | 4.1 ± 1.9 | 4.0 ± 1.9 | 0.345 | 0.058 |  | 4.0 ± 1.8 | 4.1 ± 1.9 | 0.624 | 0.034 |
| Pathological stage |  |  | 0.022 | 0.174 |  |  |  | 0.968 | 0.018 |
| Stage I | 77 (20.5) | 246 (27.9) |  |  |  | 72 (23.2) | 141 (22.7) |  |  |
| Stage II | 132 (35.1) | 286 (32.4) |  |  |  | 106 (34.1) | 217 (34.9) |  |  |
| Stage III | 167 (44.4) | 350 (39.7) |  |  |  | 133 (42.8) | 264 (42.4) |  |  |
| Tumor differentiation |  |  | 0.105 | 0.098 |  |  |  | 0.726 | 0.031 |
| Well/moderately differentiated | 308 (81.9) | 756 (85.7) |  |  |  | 260 (83.6) | 527 (84.7) |  |  |
| Poorly differentiated | 68 (18.1) | 126 (14.3) |  |  |  | 51 (16.4) | 95 (15.3) |  |  |
| Perineural invasion |  |  | 0.832 | 0.019 |  |  |  | 0.611 | 0.042 |
| Yes | 54 (14.4) | 121 (13.7) |  |  |  | 45 (14.5) | 81 (13.0) |  |  |
| No | 322 (85.6) | 761 (86.3) |  |  |  | 266 (85.5) | 541 (87.0) |  |  |
| Lymphovascular invasion |  |  | 0.243 | 0.076 |  |  |  | 0.407 | 0.066 |
| Yes | 54 (14.4) | 104 (11.8) |  |  |  | 32 (10.3) | 77 (12.4) |  |  |
| No | 322 (85.6) | 778 (88.2) |  |  |  | 279 (89.7) | 545 (87.6) |  |  |

NLR: neutrophil‑to‑lymphocyte ratio; PLR: platelet‑to‑lymphocyte ratio; ASA: American Society of Anesthesiologists; CEA: carcinoembryonic antigen.

**SUPPLEMENTARY FIGURE 8** Love plot of standardized mean differences for baseline covariates before and after propensity score matching.
